# Therapeutical and Pharmaceutical Notes

**Published:** 1901-05

**Authors:** W. J. Martin

**Affiliations:** Kankakee, Ill.


### THERAPEUTICAL AND PHARMACEUTICAL NOTES.

UNDER THE DIRECTION OF W. J. MARTIN, M.D.C.,

KANKAKEE, ILL.

---

THE EPHEMERIS. We have received No. 3, vol. vi., of the *Ephemeris*, a journal devoted to the advancement of pharmacy, materia medica, and other collateral information of value to the pharmacist, and practitioners of both human and veterinary medicine. This excellent little volume contains in a concise form a fund of information on the chemical composition, therapeutical and physiological action of many of the most important new drugs and chemicals that have appeared during the year 1900, arranged in alphabetical order. It will be sent free to any applicant by addressing its author, E. H. Squibb, M.D., New York.

TREATMENT OF WOUNDS OF THE HEART BY SUTURE. Elsberg (*Journal of Experimental Medicine*, September and November, 1899) has conducted a number of experiments on dogs and rabbits by which he has found that their hearts could be easily grasped with a forceps and the needle and suture readily passed. He infers that the difficulties of this procedure in the human heart are not as great as has been supposed.

Some patients it is known have recovered from wounds of the heart as large as three centimetres without any local treatment, and suture of the heart has been performed nine times; four recovered entirely, and four died of complications referable to other organs.

Elsberg summarizes his conclusions thus: 1. Suture of a heart wound as a final resort may often be found worthy of consideration and justifiable. 2. There is no danger of sudden arrest of the action of the heart while being manipulated, unless Kronecker's co-ordination centre be injured. 3. The suture should be of silk and interrupted; it should be applied so that the epicardium and superficial layers of the myocardium shall be the only ones penetrated, and tied when possible during diastole. 4. Each case must be considered by itself for symptoms which would justify operative interference.—*Thera. Gaz.*

TANNOCREOSOFORM. This is a combination of tannin, creosote, and formaldehyde, which occurs as a non-toxic, brownish, odorless, and tasteless powder, insoluble in water and glycerin, dissolved by dilute alkalies. It is an active antiseptic, suitable both for internal and external use. In the latter case it may be given in daily doses of 5 grammes.

AMYL SALICYLATE. B. Lyonnet suggests that amyl salicylate may be usefully substituted for methyl-salicylate in medicine. Internally, it is practically non-toxic; applied to the skin, it is non-irritant, and is readily absorbed. It has a much less powerful odor than the methyl ester, and combines with the action of the salicylic acid it contains the sedative effect of amylic derivatives.

ICHTHARGAN. This new silver compound is, according to Aufrecht, a powerful antiseptic. It forms a brown, odorless, stable powder, which is soluble in water and contains 30 per cent. of silver. It is also soluble in glycerin and in dilute alcohol. The aqueous solution is darkened by direct light. It should, therefore, be stored in orange-glass bottles.—*Pharm. Journ.*

ETIOLOGY OF YELLOW FEVER. Reed, Carroll, and Agramonte (*Journal American Medical Association*), as a result of their well-known experiments in Cuba concerning the etiology of yellow fever, reached the following conclusions:

1. The mosquito—culex fasciatus—serves as the intermediate host for the parasite of yellow fever.

2. Yellow fever is transmitted to the non-immune individual by means of the bite of the mosquito that has previously fed on the blood of those sick with the disease.

3. An interval of about twelve days or more after contamination appears to be necessary before the mosquito is capable of conveying the disease.

4. The bite of the mosquito at an earlier period after contamination does not appear to confer any immunity against a subsequent attack.

5. Yellow fever can also be experimentally produced by the subcutaneous injection of blood taken from the general circulation during the first and second days of the disease.

6. An attack of yellow fever produced by the bite of the mosquito confers immunity against the subsequent injection of the blood of an individual suffering from the non-experimental form of this disease.

7. The period of incubation in thirteen cases of experimental yellow fever has varied from forty-one hours to five days and seventeen hours.

8. Yellow fever is not conveyed by fomites, and hence disinfection of articles of clothing, bedding, or merchandise supposedly contaminated by contact with those sick with the disease is unnecessary.

9. A house may be said to be infected with yellow fever only when there are present within its walls contaminated mosquitoes capable of conveying the parasite of the disease.

10. The spread of yellow fever can be most effectually controlled by measures directed to the destruction of mosquitoes and the protection of the sick against the bites of these insects.

11. While the mode of propagation of yellow fever has now been definitely determined, the specific cause of this disease remains to be discovered.

Icthoform and its Employment in Medical Therapy. The studies in the bactericidal properties of this drug, especially those made by Rabor and Galli-Valere, are reviewed at length by R. Pollacco (*Boll. Clin. Scien. della Peramb. di Milano*), who proceeds to give an account of his own investigations, which are purely clinical. He finds that icthoform is the most energetic and at the same time the most harmless of intestinal antiseptics which modern therapy places at our disposal. It exercises no injurious effect either upon the heart, the liver, or kidneys even in the most liberal doses. Pollacco thinks that icthoform has a specific action in diarrhœa of tubercular patients, and is superior to any other antiseptic known in typhoid and in affections due to the bacterium coli. It cures all kinds of diarrhœa, and where opportunity presents itself the author would recommend its use in the dysenteric forms. Icthoform combines the strongly analgesic, astringent, and antiseptic property of icthol with the energetic antiseptic action of formicaldehyde.

The Salicylates and Alkalies in Rheumatic Fever. A. Haig (*Medical Record*) asserts that while the arthritis of rheumatic fever is benefited by either the salicylates or alkalies alone they do harm if given together, since in combination they no longer act as solvents of uric acid. Conditions which raise the alkalinity of the blood reduce the value of the salicylates, hence they act less effectively in hot weather or in hot climates which conduce to free perspiration. Salicylates act best when the blood is active and are most effective when fever is high and the urine very acid.—*Medical Standard.*

